# Contributions of hemispheric dynamics in visual word recognition: uncovering familiarity effects through lateralized priming

**DOI:** 10.3389/fpsyg.2024.1475475

**Published:** 2024-12-17

**Authors:** Sangyub Kim, Kichun Nam

**Affiliations:** ^1^Department of Psychology, Chonnam National University, Gwangju, Republic of Korea; ^2^School of Psychology, Korea University, Seoul, Republic of Korea

**Keywords:** visual word recognition, stimulus onset asynchrony (SOA), hemispheric specialization, lexical decision task (LDT), hemispheric dynamics, familiarity

## Abstract

**Introduction:**

This investigation aimed to explore interhemispheric interactions in visual word processing with a focus on proficiency development. Given the asymmetrical specialization in visual word processing across hemispheres, the study hypothesized that the primary hemisphere predominantly regulates interhemispheric interactions. The familiarity effect, serving as a measure of visual word processing proficiency, was examined to determine how proficiency influences these interactions.

**Methods:**

A primed-lateralized lexical decision task with a stimulus onset asynchrony (SOA) of 100 ms was employed. The task involved presenting primes and targets in parafoveal visual fields (left visual field/right visual field) to assess behavioral responses. By manipulating prime and target visual field locations, the study aimed to evaluate both inter- and intrahemispheric interactions during visual word processing.

**Results:**

The findings revealed a significant interhemispheric familiarity effect in response times when the left visual field (LVF)/right hemisphere (RH) served as the prime and the right visual field (RVF)/left hemisphere (LH) as the target. Additionally, a significant intrahemispheric familiarity effect was observed within the LVF/RH condition, suggesting a prominent role of the RH in visual-perceptual processing during the development of visual word recognition proficiency.

**Discussion:**

These results provide compelling evidence for asymmetric specialization between the hemispheres in visual word processing. The significant inter- and intrahemispheric familiarity effects underscore the importance of RH visual-perceptual processing in proficiency development. These insights enhance our understanding of interhemispheric dynamics in the evolution of visual word recognition proficiency, highlighting the complex coordination between hemispheres in facilitating fluent visual word processing.

## Introduction

Cognitive proficiency is a critical area of exploration in cognitive psychology, largely due to the unresolved complexities inherent in its developmental processes. The human brain constantly strives for enhanced performance (Draganski et al., 2006). Thus, repeated engagement in specific cognitive activities prepares individuals for particular cognitive tasks (i.e., Jaeggi et al., 2008). This preparation is facilitated by an intrinsic mechanism known as brain plasticity (Zatorre et al., 2012). However, the development of cognitive proficiency is not only associated with neuronal maturation but also with the interhemispheric interaction between the brain’s two hemispheres (Paus et al., 1999). These hemispheres possess mechanisms that enable lateralization (Toga and Thompson, 2003), thereby potentially allowing for efficient processing (Gazzaniga, 2000). This assumption allows us to investigate the changes in interhemispheric interactions during the development of cognitive proficiency. Hence, elucidating the dynamic mechanisms at play in the brain during this developmental phase offers valuable insights into the understanding of how cognitive proficiency evolves, particularly in terms of interhemispheric interaction, as the current investigation encompasses both macro halves of the brain.

Among the myriad cognitive activities, reading emerges as a critical function in cognitive processing, necessitating early acquisition to facilitate effective communication. To comprehend the development of reading, it is vital to investigate the underlying neural mechanisms during this developmental phase. A promising approach to understanding the neural alterations accompanying reading proficiency is the investigation of visual word processing, particularly through the lens of visual familiarity with words. Word familiarity, defined by the extent to which a word is recognizable to an individual, as evidenced by Kim et al. (2020) which found a strong positive correlation between word familiarity and word frequency. Word familiarity plays a crucial role in visual word recognition, facilitating more rapid lexical access. Familiar words are generally processed more efficiently than unfamiliar ones (Kim et al., 2020; Kim et al., 2022a; Kim et al., 2023b; Kuperman and Van Dyke, 2013; Mahboob, 2006), as their recognition demands reduced cognitive resources. While word frequency—the statistical prevalence of a word within a language corpus—has traditionally served as a basis for research in visual word recognition, familiarity is more suitable in investigation of proficiency in visual word recognition. Frequency reflects objective repetition, yet familiarity encompasses both frequency and the subjective ease of recognition, embedding a cognitive dimension into word processing. Notably, words classified as high frequency in large corpora, such as the Corpus of Contemporary American English (COCA), may not be perceived as highly familiar in daily language use. For example, while “paradigm” appears approximately 3.5 times per million words in COCA, “tsunami,” appearing around once per million words, is often more immediately familiar to speakers. This contrast indicates the importance of integrating familiarity measures to accurately evaluate proficiency in word recognition, aligning with an individual’s real-world language experience. Thus, proficiency in visual word recognition is significantly enhanced by familiarity, which signals frequent exposure and may reveal the neural dynamics underlying the development of recognition proficiency, particularly regarding hemispheric interactions.

In this regard, Kim et al. (2022a) assessed the impact of word familiarity on bilateral redundancy gain (BRG) by presenting words at both unilateral and bilateral parafoveal visual fields. BRG, which indicates improved performance through bihemispheric rather than unihemispheric processing, was evaluated by comparing behavioral responses for words presented simultaneously in the left and right visual fields with behavioral performance for words presented unilaterally. Their findings revealed a significant BRG effect on RTs only for the most familiar words, with no observed BRG for words of lower familiarity levels. Their finding suggests a facilitative interaction for familiar word recognition, contrasting with less familiar word recognition. Further, Kim et al. (2023b) explored word familiarity effects at four levels by presenting words centrally and recording electroencephalography (EEG) responses. Behavioral analyses indicated enhanced speed and accuracy for highly familiar words, while EEG event-related potentials (ERPs) highlighted an asymmetry in the N100 and N400 components, reflecting hemispheric differences in familiarity processing. Granger causality analyses showed a stronger right-to-left hemisphere transfer during N100 for familiar words and a weaker left-to-right transfer during N400, suggesting familiarity-dependent shifts in interhemispheric dynamics for visual word recognition. The observed familiarity effect, marked by faster and more accurate responses in lexical decisions, including the concomitant left and right hemisphere interactions for familiar words, suggests that this effect mirrors alterations in information processing as proficiency increases in visual word processing.

This study investigates the role of the right hemisphere (RH) in the familiarity effect during visual word recognition, highlighting its specialized function in visual-perceptual processing. This function is regarded as secondary to the left hemisphere’s primary responsibilities in language processing. As the findings of Kim et al. (2023b) suggest a facilitative role of the RH in augmenting LH function in recognizing highly familiar words, their findings support the findings of Nowicka and Tacikowski (2011) concerning the directional flow of asymmetric transfer from the RH to the LH in language processing. Nowicka and Tacikowski (2011) posited that LH lateralization for language processing may stem from an asymmetrical facilitative transfer originating from the non-dominant hemisphere (indicating RH) to the dominant hemisphere (indicating LH) during language processing (Nowicka and Tacikowski, 2011). They suggested that the asymmetric transfer from the RH to the LH in language processing may signify greater left-lateralization in the visual processing of highly familiar words. This notion finds support in previous evidence indicating that hemispheric lateralization could be mediated by the corpus callosum—a structural conduit connecting the hemispheres, facilitating interactive processes conducive to hemispheric specialization. Thus, the asymmetric transfer from the RH to the LH may underpin proficient visual word recognition processing.

Furthremore, findings from studies employing parafoveal and foveal lexical decision tasks (Kim et al., 2020; Kim et al., 2022a) indicate that responses to right parafoveal words were characterized by slower RTs and increased inaccuracies compared to responses to foveal words. Despite right parafoveal words being projected to the language-dominant hemisphere (left hemisphere), more efficient processing was observed in foveal vision (with projection to both hemispheres). This efficiency may stem from the RH’s capacity to facilitate LH processing, as evidenced by asymmetric facilitation from the RH to the LH. These findings lend support to the notion that asymmetric transfer from the RH to the LH supports proficient visual word recognition processing.

### Dual route cascaded model of visual word recognition

Modulations in interhemispheric interaction between the LH and RH contingent upon word familiarity may align with principles posited in the dual route cascaded (DRC) model. The DRC model of visual word recognition offers a theoretical framework delineating two distinct pathways through which words are processed and identified (Coltheart et al., 2001). These pathways operate concurrently and dynamically interact during reading endeavors. Originally formulated to investigate how proficient readers swiftly and accurately recognize words, the model accommodates diverse factors including word frequency, regularity, and familiarity (Coltheart et al., 2001). The first pathway, known as the lexical route or the “direct” route, entails direct access to stored representations of familiar words within long-term memory. If the word corresponds to a familiar and frequently encountered lexical item, its identification is promptly and effortlessly accomplished via the lexical route. This pathway is characterized by holistic processing, wherein the word is apprehended as an integral entity and the necessity for decomposition into constituent elements (such as letters or phonemes). Conversely, the second pathway, termed the non-lexical route or the “indirect” route, engages in sequential processing of individual letters or graphemes to derive their phonological representations. The indirect route typically operates when confronted with unfamiliar or irregular words, as well as nonwords lacking stored representations within the mental lexicon.

Considering that the LH predominantly manages both lexical and sublexical processing during reading (Joubert et al., 2004), whereas the RH specializes in visual-perceptual processing (Baumgaertner et al., 2013), it is plausible that the interplay between these hemispheres varies contingent upon the familiarity of the processed words. In instances where words are highly familiar and commonly encountered, such as frequently used words in one’s native language, the LH may heavily rely on lexical processing (Kim et al., 2023c; Weems and Zaidel, 2005), accessing stored representations in the mental lexicon directly. In the study by Kim et al. (2023c), identical prime and target words were presented parafoveally in a sequential format, with intervals between them, replicating the procedure used in the current study. This setup enabled a comparison of repetition priming across intrahemispheric locations in both the left and right hemispheres. The authors hypothesized that if intrahemispheric repetition priming for parafoveally presented words were governed primarily by episodic memory, the effect would be uniform across hemispheres. However, if the LH is indeed specialized for lexical processing, greater intrahemispheric repetition priming would be observed in the RVF/LH compared to the LVF/RH. Their findings aligned with this latter hypothesis, showing stronger intrahemispheric repetition priming for words in the RVF/LH, suggesting contributions from both lexical processing and episodic memory. In contrast, nonwords—lacking lexical representation—exhibited repetition priming effects governed primarily by episodic memory, resulting in similar intrahemispheric repetition priming intensities across the RVF/LH and LVF/RH. This aligns with Logan; Logan’s (1988; 1990) findings that facilitative priming for nonwords relies on memory for physical attributes, supporting a distinct episodic mechanism for nonwords.

Reflecting these hemispheric asymmetries in lexical processing, Kim et al. (2023c) identified distinct interhemispheric priming effects for lexical decisions, underscoring the LH’s role in lexical processing and the RH’s support in visual attribute and contextual cue integration. For words, priming from the left to the right hemisphere was stronger, reflecting the left’s lexical specialization. Conversely, for nonwords, priming from the right to the left hemisphere dominated, as nonwords lack lexical representation, enabling the RH’s visual expertise to drive familiarity judgments. These results suggest interhemispheric interaction manifest as efficient coordination of information between hemispheres to facilitate swift and precise word recognition.

Considering the explanation above, when faced with less familiar or novel words, the LH may lean toward sublexical processing via the assumed sublexical route posited in the DRC model, encompassing phonological decoding or grapheme-to-phoneme conversion. During such instances, the RH may still contribute to visual feature processing and offer contextual assistance, albeit with a divergence in the relative contributions of lexical and sublexical processing mechanisms. This distinct pattern of interhemispheric interaction might be discernible through repetition priming effects, particularly evident in parafoveal vision where identical stimuli are rapidly presented in succession.

### The current study

We expected that disparities in the familiarity effect across cerebral hemispheres will manifest distinctly in RTs and ACC, reflecting their processing specializations. Previous research indicates the LH dominance in lexical and sublexical processing during reading (Joubert et al., 2004) and the RH specialization in visual-perceptual processing (Baumgaertner et al., 2013; Kim et al., 2024a). The RH’s proficiency in visual-perceptual processing is expected to enhance familiarity effects during parafoveal word recognition, as the familiarity experienced in the RH is linked to perceptual learning mechanisms (Cohen and Dehaene, 2004; Dehaene et al., 2010), whereby familiarity is established through repeated exposure and visual processing pathways. Specifically, we expected that familiar words presented in the left visual field (LVF)/RH will yield significant familiarity effects in RTs, attributable to the RH’s superior visual-perceptual capabilities, which concurrently support the analytical processing of the LH.

We further expected that when primes are presented in the right visual field (RVF)/LH, significant intra- and inter-hemispheric familiarity effects will be observed in ACC. This finding suggests the LH’s role as the decision-making hemisphere, led by its superior lexical and sublexical processing capabilities in lexical tasks. We anticipated this pattern to emerge despite the absence of significant familiarity effects in RTs when primes are displayed in the RVF/LH. This suggests that LH activation is more closely associated with ACC in decision-making rather than the speed of lexical processing, which is predominantly influenced by visual-perceptual attributes in the RH. We proposed that the inherent asymmetry in hemispheric processing will enhance visual-perceptual familiarity effects in the RH while simultaneously facilitating the LH’s processing capabilities for lexical decisions.

Moreover, we anticipated a differential lexicality effect, reflecting the distinct processing of words and nonwords across the left and right hemispheres, attributable to their specialized functions. Specifically, we expect that the LH, known for its dominance in lexical processing, will exhibit faster RTs for words presented in the RVF/LH. This expectation is supported by evidence indicating that lexical access is more efficient in the LH, facilitating the rapid recognition of words relative to nonwords. Conversely, the RH, which specializes in visual-perceptual processing, is anticipated to yield higher ACC in differentiating nonwords from words when nonwords are presented in the LVF, projecting to the RH. This expectation aligns with prior research demonstrating that the RH’s visual-perceptual capabilities enhance its ability to identify “non-wordness,” an essential skill for distinguishing nonwords from words based on visual features.

Therefore, we hypothesized that the familiarity effect—reflected in faster RTs and higher ACC for more familiar words—would be observed when the prime was presented in the LVF/RH and the target in the RVF/LH, suggesting interhemispheric interaction from RH to LH. Additionally, we expected this familiarity effect to manifest when both prime and target were presented in the LVF/RH, indicative of intrahemispheric processing within RH. Furthermore, we hypothesized significant intra- and interhemispheric lexicality effects, with faster RTs for words relative to nonwords when the prime was displayed in the RVF/LH, and higher accuracy (ACC) for words over nonwords when the prime was presented in the LVF/RH.

## Methods

### Participants

A total of 48 participants were recruited from Korea University, South Korea, including 22 males and 26 females, with an average age of 25.63 (SD = 3.50). Participants reported an average of 18.63 years of education (SD = 3.50). All participants were strongly right-handed, as assessed by the Edinburgh Handedness Inventory (*M*: 8.06, SD: 1.79). All participants’ data were included for final analysis as participants adhered to the experimental protocol without any complications. The study was conducted in accordance with the ethical principles delineated in the 1964 Declaration of Helsinki, and approved by the Institutional Review Board of Korea University (KUIRB-2018-0086-01). Prior to participation, all individuals were thoroughly briefed on the study’s ethical guidelines and provided informed consent. Participants were compensated with a payment of seven thousand won for their involvement. All reported normal or corrected-to-normal vision, with no history of dyslexia, neurological impairments from brain damage or stroke, sensory organ impairments, mental illness diagnoses, or substance abuse or addiction.

### Experimental task and procedure

To examine the alteration of interhemispheric processing according to the familiarity of words, we employed the visual half-field presentation paradigm. This paradigm involves the presentation of visual stimuli in the left and/or right parafoveal fields, anchored by a central fixation point. Specifically, the left parafoveal presentation is projected to the RH, initiating processing within the RH, while the right parafoveal presentation is directed to the LH, initiating processing within the LH. This paradigm offers a versatile platform to investigate hemispheric interactions, allowing for sequential presentation of stimuli. Sequential presentation in the parafoveal visual fields, following a particular order such as LVF to RVF, orchestrates a sequence of hemispheric activation corresponding to the order of stimuli presentation. For instance, a sequence presentation of LVF-RVF enables the evaluation of interhemispheric interaction from the RH to LH in visual word processing. We expected that if a specific pattern of interhemispheric activation correlates with reading proficiency, examining the visual familiarity effect during word recognition could aid investigate developmental changes.

In addition, we manipulated the SOA between the prime and target, specifically at intervals of 100 ms. We particularly chose the SOA 100 ms to investigate the effect of the RH’s primary visual-perceptual processing on the asymmetric transfer from the RH to the LH. based on Holcomb and Grainger (2007). Their bimodal interactive model argues that the processing stages of word recognition can be divided into three stages: the processing stages of visual features, prelexical, and lexical level representation. This model posits that information processing related to visual features transpires up to approximately 100 ms post-stimulus presentation, with prelexical and lexical processing. Therefore, the sequential presentation at the left and right parafoveal vision, coupled with different SOAs between the prime and target, may induce a differential pattern of interhemispheric interaction. This is attributed to the distinct specialization of visual word processing across the two hemispheres. Taking into account the distinctive specialization of the two hemispheres (Barca et al., 2011; Brysbaert, 2004; Chu et al., 2020; Kim et al., 2022b; Kim and Nam, 2023a,b; Mohr et al., 1994; Van der Haegen et al., 2009), characterized by primary visual-perceptual processing employing global attributes in the RH and primary lexical processing in the LH, our anticipation was the emergence of significant interhemispheric interaction from the RH to LH and/or significant intrahemispheric interaction within the RH at an SOA of 100 ms in visual word recognition. The expectation is grounded in the understanding that primary visual-perceptual processing using global attributes may precede lexical processing in the RH (Hellige, 1996; Lamb and Robertson, 1988; Lamb et al., 1989, 1990).

In alignment with the objectives of the current study, the experimental design encompassed several conditions to probe the underlying mechanisms of visual word recognition. These conditions were organized into the prime visual field (PVF) with two levels (LVF/RVF), the target visual field (TVF) with two levels (LVF/RVF), and the familiarity condition with four levels (F1/F2/F3/F4). The PVF and TVF conditions, involving stimuli presentation in either the left or right visual field, were instrumental in the examination of intra/interhemispheric processing within the parafoveal visual field. These conditions were constructed to isolate the neural pathways engaged in visual word recognition. Additionally, the familiarity condition was introduced to investigate intra/interhemispheric information processing as a function of word familiarity. This aspect of the design allowed for an exploration of how familiarity with a given word might influence the interhemispheric processing. Furthermore, a lexicality condition was incorporated to discern the differences in responses between word and nonword stimuli, reflecting the presence or absence of processing experience. This added dimension enabled a more comprehensive investigation into information processing based on word familiarity, thereby enriching our understanding of the complex interplay between intra-/inter-hemispheric processing and word familiarity. The integration of these conditions provided a framework for dissecting the hemispheric mechanisms underlying visual word recognition.

**FIGURE 1:**
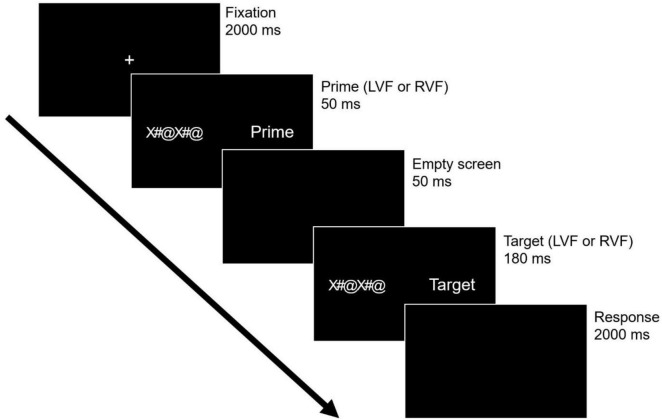
Description of experimental procedure in the primed-lateralized lexical decision task.

Thus, we utilized a primed-lateralized lexical decision task in the current study to explore the intra/interhemispheric processing of visual words (Figure 1) (Kim et al., 2023c; Kim et al., 2024b), employing identical stimuli presented at the parafoveal vision. Participants were thoroughly briefed on the task procedures before initiating the experiment by pressing the space bar on the keyboard. The task commenced with a central fixation point displayed for 2000 ms to ensure proper fixation. Following this, a prime was briefly presented for 50 ms in either the LVF or RVF, designed to initially propagate to the contralateral hemisphere. A mask (“X#@X#@”) was simultaneously displayed in the opposite visual field to that of the prime. A subsequent empty screen interval of 50 ms was provided to facilitate interaction with the target, which was then presented at the parafoveal visual field for 180 ms, accompanied by the mask. Participants were instructed to respond within a 2000 ms blank window to categorize the stimulus as either a word or nonword. In addition, a Latin square design was implemented in the experiment to ensure that participants were not repeatedly exposed to the same stimuli across different visual field conditions. Given the two levels in both the PVF and TVF conditions, a total of 600 stimuli (300 words and 300 nonwords) were divided into four lists of 150 items each (comprising 75 words and 75 nonwords). These lists were structured to avoid any overlap between stimuli, ensuring that each list remained distinct for presentation to participants.

To do so, participants were positioned with their chin resting on a chinrest, maintaining a distance of 65 cm between the nasion and the monitor. This fixed position was maintained throughout the experimental task to minimize variability. Stimuli were presented on an LG monitor with the capability to display RGB colors. Participants’ responses were captured via a keyboard strategically positioned in front of them. The instructions were provided for response mechanisms: participants were directed to use their right index finger to press the “slash” button for word responses, and their left index finger to press the “z” button for nonword responses. The assignment of response hand was counterbalanced across participants to mitigate potential biases. The experimental procedure was conducted using Experimental Psychology Software (E-prime), a specialized tool chosen for its precision and reliability in psychological research. This rigorous approach to experimental design ensured the integrity of the data collected, providing a robust foundation for the subsequent analysis and interpretation of the intra/interhemispheric processing dynamics under investigation.

### Materials

To enhance ecological validity, the current study utilized morphologically complex words, which are common in Korean and are typically separated by spaces to facilitate language processing in everyday contexts (Kim et al., 2020). The words employed in this study were selected in a non-biased manner, adhering to a predetermined distribution sourced from mediums: newspapers, movies, published papers/articles, and internet blogs. The objective was to explore the cognitive processing of word stimuli encountered in everyday contexts. Words with possible noun-verb ambiguities were excluded in the final stimuli set.

The current study explored an array of lexical variables that encompass diverse dimensions of word characteristics, derived from the Kang and Kim (2009) corpus. These variables included sublexical/lexical related to word length, encompassing factors such as number of stroke, number of phoneme, number of syllable, and number of morpheme. Furthermore, the analysis investigated the semantic aspect by examining the number of meanings. Additionally, frequency-related variables are integrated into the investigation, covering aspects of first syllable frequency and familiarity. Notably, familiarity, as previously assessed in Kim et al. (2020), is a critical component of the present investigation. In Kim et al. (2020), they evaluated word familiarity by requiring participants to rate their familiarity with each word on a Likert scale ranging from 1 (least familiar) to 7 (most familiar), following the methodology established by Kreuz (1987). They assessed a total of 300 Korean words, which we incorporated into the current research by categorizing them into four distinct lists based on their familiarity ratings. Each list comprises 75 words, resulting in a total of 300 words. In the familiarity condition, factors such as physical length (number of strokes, phonemes, syllables, and morphemes), semantics (number of objective meanings), and frequency (log-transformed first syllable frequency) other than familiarity were carefully matched across familiarity levels: F1 (least familiar), F2 (moderately unfamiliar), F3 (moderately familiar), and F4 (most familiar)^1^. The mean familiarity values ( ± standard errors) across familiarity levels were: F1 = 4.212 ± 0.085, F2 = 4.609 ± 0.105, F3 = 5.098 ± 0.087, and F4 = 5.464 ± 0.091, indicating a significant familiarity gradient across levels, [*F*(3, 296) = 35.415, *p* < 0.001, $\eta_{p}^{2}$ = 0.264]. Bonferroni post-hoc tests confirmed that each pairwise comparison between familiarity levels was statistically significant. The corpus encompassed a total of 300 extracted words, alongside an equivalent count of 300 nonword stimuli, ensuring a balanced dataset for analysis. To this end, we employed nonword stimuli comprising syllables that are dissimilar to words, ensuring a contrast in phonetic composition.

## Results

Data were collected for both RTs and accuracy (ACC) in the primed-lateralized lexical decision task. A preprocessing analysis revealed that the ACC for words and nonwords for all participants fell within 3 standard deviations, with the exception of 5 participants. Consequently, the data from these 5 participants were excluded from the final analysis to ensure the integrity and robustness of the results.

1. Familiarity effect on the interhemispheric interactions in RTs and ACC

**FIGURE 2:**
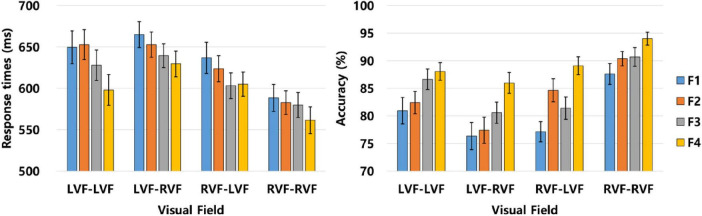
Graphical representation depicting the familiarity effect assessed by both RTs **(left panel)** and ACC **(right panel)**. The bars are accompanied by lines indicating standard errors. F1, least familiar; F2, moderately unfamiliar; F3, moderately familiar; F4, most familiar; LVF, left visual field; RVF, right visual field.

The behavioral responses for the familiarity effect, including both RTs and ACC, is provided in Figure 2 and Table 1. A mixed-effect regression analysis was conducted using the R software to examine the influence of familiarity on RTs and ACC for words (R Core Team, 2012). The analytical model was constructed to encompass both fixed and random effects. The fixed effects included variables such as familiarity (F1 / F2 / F3 / F4), PVF (LVF / RVF), TVF (LVF / RVF), and their two-way interactions (familiarity × PVF, familiarity × TVF, PVF × TVF). Additionally, a three-way interaction (familiarity × PVF × TVF) was incorporated to capture the interplay between these factors. Random effects were also included in the model to account for participant and item variability.

**TABLE 1:** Comprehensive summary of the behavioral responses observed in terms of RTs and ACC within each experimental condition for the familiarity effect in experiment.

		Level of familiarity							
		**F1 (least familiar)**		**F2 (moderately unfamiliar)**		**F3 (moderately familiar)**		**F4 (most familiar)**	
PVF	TVF	RTs	ACC	RTs	ACC	RTs	ACC	RTs	ACC
LVF	LVF	650 (131)	0.809 (0.158)	653 (118)	0.824 (0.133)	628 (120)	0.866 (0.122)	598 (121)	0.881 (0.105)
	RVF	665 (102)	0.764 (0.162)	653 (100)	0.774 (0.156)	639 (94)	0.806 (0.126)	630 (102)	0.860 (0.124)
RVF	LVF	637 (123)	0.771 (0.119)	624 (103)	0.846 (0.137)	603 (102)	0.814 (0.133)	605 (96)	0.891 (0.107)
	RVF	588 (106)	0.876 (0.124)	583 (94)	0.904 (0.085)	580 (99)	0.907 (0.111)	561 (106)	0.940 (0.075)

The values within brackets represent the corresponding standard deviations. LVF, left visual field; RVF, right visual field; PVF, prime visual field; TVF, target visual field.

The initial analysis was conducted on RTs, utilizing the lmer function in R. The results showed significant main effects across several dimensions, including familiarity [β = −6.774, SE = 2.058, *t* = −3.291, *p* = 0.001], PVF [β = −20.988, SE = 1.832, *t* = −11.456, *p* < 0.001], and TVF [β = −6.560, SE = 1.833, *t* = −3.579, *p* < 0.001]. Noteworthy were the two-way interaction effects observed between PVF and TVF [β = −15.014, SE = 1.832, *t* = −8.197, *p* < 0.001] and between familiarity and PVF [β = 2.306, SE = 1.157, *t* = 1.993, *p* = 0.046]. Conversely, the two-way interaction effect between TVF and familiarity and the three-way interaction effect between the factors were not significant [β = −0.086, SE = 1.157, *t* = −0.075, *p* = 0.941 for familiarity × TVF; β = 0.169, *SE* = 1.157, *t* = 0.146, *p* = 0.884 for familiarity × PVF × TVF].

The significant main effect of familiarity pointed to faster RTs for more familiar words. Furthermore, the significant main effects of PVF and TVF indicated faster RTs when the prime and target were presented at the RVF compared to the LVF. The two-way interaction effect between PVF and TVF revealed a pattern: the RVF target elicited slower responses than the LVF target when the prime was presented at the LVF [β = 8.069, SE = 2.696, *t* = 2.993, *p* = 0.003], while the opposite was significant when the prime was presented at the RVF [β = −21.180, SE = 2.474, *t* = −8.560, *p* < 0.001]. The two-way interaction effect between familiarity and PVF found a significant familiarity effect when the prime was presented at the LVF [β = −8.583, SE = 2.449, *t* = −3.505, *p* < 0.001], meaning faster responses for familiar words when the prime was given at the LVF. This effect was not observed when the prime was presented at the RVF [β = −4.132, SE = 2.314, *t* = −1.786, *p* = 0.075].

Following the analysis of RTs, ACC was examined using the glmer function in R. The results showed significant main effects for familiarity [β = 0.157, *SE* = 0.035, *z* = 4.498, *p* < 0.001], PVF [β = 0.249, SE = 0.028, *z* = 8.889, *p* < 0.001], and TVF [β = 0.107, SE = 0.028, *z* = 3.831, *p* < 0.001]. Two-way interaction effects were observed between PVF and TVF [β = 0.260, SE = .028, *z* = 9.305, *p* < 0.001] and between familiarity and PVF [β = 0.049, SE = 0.018, *z* = 2.736, *p* = 0.006], with a significant three-way interaction effect between the factors [β = −0.041, SE = 0.018, *z* = −2.326, *p* = 0.020]. Conversely, the two-way interaction effect between familiarity and TVF was not significant [β = −0.012, SE = 0.018, *z* = −0.674, *p* = 0.500].

The significant main effect of familiarity pointed to higher ACC for familiar words, while the main effects of PVF and TVF indicated higher ACC when both prime and target were presented at the RVF. The two-way interaction effect between PVF and TVF revealed that the RVF target was responded to less accurately when the prime was presented at the LVF [β = −0.152, SE = 0.036, *z* = −4.253, *p* < 0.001], and more accurately when the prime was presented at the RVF [β = 0.388, SE = 0.042, *z* = 9.206, *p* < 0.001]. Furthermore, the two-way interaction effect between familiarity and PVF indicated that the familiarity effect was significant when the prime was presented at the LVF [β = 0.115, *SE* = 0.038, *z* = 3.029, *p* = 0.002] and at the RVF [β = .213, SE = 0.040, *z* = 5.337, *p* < 0.001]. Additionally, the three-way interaction effect indicated a significant familiarity effect when the prime was presented at the LVF and the target at the RVF [β = 0.152, SE = .045, *z* = 3.367, *p* < 0.001], but not when both were presented at the LVF [β = 0.086, SE = 0.045, *z* = 1.932, *p* = 0.053]. The effect was significant for both left and right visual field presentations of the target when the prime was presented at the RVF [β = .267, SE = .048, *z* = 5.554, *p* < .001 for LVF target; β = .156, SE = .050, *z* = 3.114, *p* = 0.002 for RVF target].

2. Lexicality effect on the interhemispheric interactions in RTs and ACC

**FIGURE 3:**
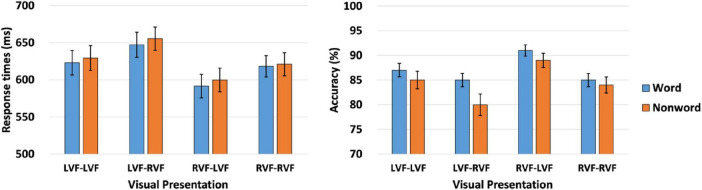
Graphical representation depicting the lexicality effect assessed by both RTs **(left panel)** and ACC **(right panel)**. The bars are accompanied by lines denoting standard errors. LVF, left visual field; RVF, right visual field.

Figure 3 and Table 2 presents the behavioral responses associated with the lexicality effect, encompassing both RTs and ACC. A mixed-effect regression analysis was conducted to investigate the lexicality effect on RTs and ACC for both words and nonwords. The model included fixed effects for lexicality (word / nonword), PVF (LVF / RVF), TVF (LVF / RVF), and their respective two-way interactions (lexicality × PVF, lexicality × TVF, PVF × TVF), as well as a three-way interaction (lexicality × PVF × TVF). Random effects were also incorporated to account for variations among participants and items.

**TABLE 2:** Comprehensive summary of the behavioral responses observed in terms of RTs and ACC within each experimental condition for the lexicality effect in Experiment.

		Word		Nonword	
PVF	TVF	RTs	ACC	RTs	ACC
LVF	LVF	628(106)	0.846(0.149)	636(111)	0.821(0.164)
	RVF	655(111)	0.832(0.105)	662(109)	0.777(0.172)
RVF	LVF	597(103)	0.903(0.081)	603(102)	0.862(0.151)
	RVF	624(97)	0.829(0.116)	635(112)	0.809(0.145)

The values within brackets represent the corresponding standard deviations. LVF, left visual field; RVF, right visual field; PVF, prime visual field; TVF, target visual field.

In the initial analysis, the lmer function in R was utilized for RTs. The results revealed significant main effects for lexicality [β = 10.562, SE = 2.388, *t* = 4.423, *p* < .001] and PVF [β = −15.276, SE = 1.326, *t* = −11.517, *p* < 0.001], while the main effect for TVF was not significant [β = −0.647, SE = 1.327, *t* = −0.487, *p* = 0.626]. Notably, significant two-way interactions were observed between all factors [lexicality × PVF: β = 5.186, SE = 1.327, *t* = 3.910, *p* < 0.001; lexicality × TVF: β = 5.618, SE = 1.327, *t* = 4.235, *p* < 0.001; PVF × TVF: β = −13.135, SE = 1.326, *t* = −9.905, *p* < 0.001]. However, the three-way interaction among all factors was not significant [β = 1.634, SE = 1.326, *t* = 1.232, *p* = 0.218].

The significant main effect of lexicality indicated more rapid RTs for words compared to nonwords. The main effect of PVF revealed quicker responses when the prime was presented in the RVF as opposed to the LVF. Furthermore, the two-way interaction between lexicality and PVF showed a significant simple main effect of lexicality, meaning faster RTs for words than for nonwords, when the prime was presented in the RVF [β = 15.768, SE = 2.647, *t* = 5.958, *p* < 0.001], but not in the LVF [β = 5.195, SE = 2.848, *t* = 1.824, *p* = 0.069]. Similarly, the interaction between lexicality and TVF revealed a significant simple main effect of lexicality, meaning faster RTs for words than nonwords, when the target was presented in the RVF [β = 16.740, SE = 2.627, *t* = 6.372, *p* < 0.001], but not in the LVF [β = 4.723, SE = 2.942, *t* = 1.605, *p* = 0.109]. The interaction between PVF and TVF indicated that the RVF target elicited faster responses than the LVF target, regardless of whether the prime was presented in the LVF [β = 12.505, SE = 1.927, *t* = 6.491, *p* < 0.001] or RVF [β = −13.412, SE = 1.818, *t* = −7.379, *p* < 0.001].

Following the analysis of RTs, ACC was examined using the generalized linear mixed-effects model (glmer function in R). The results revealed significant main effects for PVF [β = 0.145, SE = 0.019, *z* = 7.476, *p* < 0.001] and TVF [β = 0.074, SE = .019, *z* = 3.809, *p* < 0.001]. Notably, significant two-way interactions were observed between lexicality and PVF [β = −.088, SE = .019, *z* = −4.534, *p* < 0.001] and between PVF and TVF [β = 0.240, SE = 0.019, *z* = 12.389, *p* < 0.001]. However, the two-way interaction between lexicality and TVF and the three-way interaction among all factors were not significant [lexicality × TVF: β = −0.036, SE = 0.019, *z* = −1.840, *p* = 0.066; lexicality × PVF × TVF: β = −0.024, SE = 0.019, *z* = −1.249, *p* = 0.212].

The main effect of PVF indicated greater ACC for primes presented in the RVF compared to the LVF. Similarly, the main effect of TVF revealed more accurate responses for targets in the RVF than in the LVF. The two-way interaction between lexicality and PVF showed a significant simple main effect of lexicality, meaning more accurate responses for nonwords than for words, when the prime was presented in the LVF [β = 0.125, SE = 0.044, *z* = 2.831, *p* < 0.001], but not in the RVF [β = −0.035, SE = 0.047, *z* = −0.753, *p* = 0.452]. Furthermore, the interaction between PVF and TVF indicated that LVF targets were responded to more accurately than RVF targets when the prime was in the LVF [β = −0.163, SE = 0.026, *z* = −6.310, *p* < 0.001], whereas LVF targets were less accurate than RVF targets when the prime was in the RVF [β = 0.317, SE = 0.029, *z* = 11.040, *p* < 0.001].

## Discussion

In this study, we observed pronounced intra-/inter-hemispheric familiarity effects in RTs when the prime was presented at the LVF/RH. Additionally, significant intra-/inter-hemispheric familiarity effects were evident in ACC when the prime appeared at the RVF/LH, with a significant interhemispheric familiarity effect observed in cases of the LVF/RH prime and the RVF/LH target presentation. Furthermore, regarding the lexicality effect, we observed significant intra- and inter-hemispheric lexicality effects in RTs when the prime was displayed in the RVF/LH, and in ACC when the prime was presented in the LVF/RH. These findings substantiate the hypotheses proposed in the current study and are discussed within the framework of asymmetric processing specialization across the two hemispheres, with the LH predominating in sublexical and lexical processing, while the RH specializes in visual-perceptual processing (Barca et al., 2011; Brysbaert, 2004; Chu et al., 2020; Joubert et al., 2004; Kim et al., 2022b; Kim and Nam, 2023b; Van der Haegen et al., 2009).

In the investigation of the familiarity effect in RTs, noteworthy interhemispheric transfer from the RH to the LH and intrahemispheric transfer within the RH were observed, underscoring the significance of the RH visual-perceptual processing in the development of proficiency in visual word recognition with regard to intra-/inter-hemispheric interactions, as supported by Kim et al. (2023c), which evaluated intra-/inter-hemispheric repetition priming through a parafoveal presentation paradigm. Kim et al. (2023c) observed an asymmetry in interhemispheric repetition priming, with stronger priming effects when words were presented from the RVF/LH to the LVF/RH than in the opposite sequence. This asymmetry indicates differential hemispheric processing, leading to sequence-dependent priming effects in parafoveal word presentation. Similarly, the current study identified asymmetric interhemispheric and intrahemispheric familiarity effects in parafoveal repetition, suggesting that distinct processing specializations between the two hemispheres contribute to these effects. Building on finding of LH dominance in lexical and sublexical processing during reading (Joubert et al., 2004) and RH specialization in visual-perceptual processing (Baumgaertner et al., 2013), the observed interhemispheric priming from RH to LH, alongside intrahemispheric priming within RH, likely reflects intrinsic hemispheric differences in parafoveal repetition priming. This asymmetry aligns with perceptual learning mechanisms (Cohen and Dehaene, 2004; Dehaene et al., 2010), where familiarity can be formed through repeated exposure and visual processing pathways.

This is attributed to the notion that familiarity may be contingent upon the visual-perceptual attributes of an item, given that familiarity is a subtle sensation – one image may evoke a greater sense of familiarity compared to another, and specific elements within an image may appear more familiar than others (Bonifacci et al., 2015). Thus, the RH’s utilization of its visual-perceptual specialization is effective in supporting LH processing and, naturally, in its own functioning. Furthermore, with the SOA of 100 ms between the prime and target, an interval insufficient for the primed hemisphere to engage in lexical processing, it is implied that the initial visual-perceptual processing of the prime influenced the subsequent target processing in terms of visual perceptual familiarity with the words. Consequently, only the RH exhibited a significant familiarity effect in RTs, highlighting the advantages of the RH specialization in visual-perceptual processing for the familiarity effect. For these reasons, no familiarity effects were observed in RTs when the prime was presented at the RVF/LH. However, in ACC, significant intra-/inter-hemispheric familiarity effects were observed only when the prime was presented at the RVF/LH, suggesting that the LH predominantly serves as the decision-making hemisphere for lexical decisions rather than leading to stronger activation of related brain areas for rapid reaction. Thus, the LH can facilitate the RH processing, the nondominant hemisphere for language processing, as well as the LH processing itself for decision-making in words.

Furthermore, we found the significant intra-/inter-hemispheric lexicality effects in RTs when the prime was displayed at the RVF/LH, and in ACC when the prime was presented at the LVF/RH. The lexicality effect in RTs revealed faster responses to words compared to nonwords when either the prime or target was presented at the RVF/LH. The observed advantage of the LH prime may facilitate accelerated lexical access, given the LH’s dominance in lexical processing—a critical factor for differentiating between words and nonwords. Faster word recognition in the LH, stemming from its language dominance, manifests as a significant lexical effect when either the prime or target is presented in the RVF/LH. In contrast, the RH typically does not demonstrate a lexicality effect, attributable to its limited specialization in processing lexical information compared to the LH. This distinction is crucial because effective discrimination between words and nonwords fundamentally depends on the access to lexical resources for decision-making. This may account for the significant intra-/inter-hemispheric lexicality effect when the LH is primed. In contrast, the analysis of the lexicality effect in ACC demonstrated a higher ACC for nonwords compared to words when the prime was presented at the LVF/RH. This superiority in lexical decision-making for nonwords likely stems from the RH’s primary specialization in visual-perceptual processing. The superior visual-perceptual processing in the RH facilitates participants’ ability to discern “this is not a word” based on visual features, particularly for nonwords compared to pseudowords. Pseudowords exhibit orthographic (and phonological) similarities to words, whereas nonwords lack such similarities. The orthographic dissimilarity between words and nonwords facilitates accurate discrimination, with the RH’s superior visual-perceptual processing aiding in this task. While the RH may serve as the decision-making hemisphere for discriminating between nonwords and words based on visual features, rather than a locus for processing speed in RTs, this advantage likely enhances ACC in lexical decisions for nonwords without necessarily leading to expedited RTs for nonwords. One might wonder why the lexicality effect was not significant in the RTs despite the RH possessing superior visual-perceptual processing abilities compared to the LH. Given the RH’s superior capabilities, an impact on RTs might be anticipated. However, the statistical analysis of RTs, when the prime was presented in the LVF/RH and across left and right target visual fields, revealed a tendency toward faster RTs for words over nonwords. Nonetheless, this trend did not achieve statistical significance [β = 5.195, SE = 2.848, *t* = 1.824, *p* = 0.069]. This pattern suggests a potential trend of faster RTs for words, which may corroborate findings in ACC.

The findings of the present study provide empirical support for the DRC model in the domain of visual word recognition, particularly concerning hemispheric interactions. Given the dominant role of the LH in both sublexical and lexical processing during reading (Joubert et al., 2004), contrasted with the RH’s specialization in visual-perceptual processing (Baumgaertner et al., 2013), it is plausible to conjecture that the interplay between these hemispheres is contingent upon the familiarity of the processed words. In contexts involving highly familiar or high-frequency words, the LH may predominantly engage in lexical processing, while the RH assumes a supportive function, particularly in processing visual attributes and integrating contextual cues. This interhemispheric interaction likely facilitates efficient coordination of information between hemispheres (Gazzaniga, 2000), enhancing the speed and accuracy of word recognition. Conversely, when confronted with less familiar or novel words, the LH may shift toward sublexical processing via the proposed sublexical route outlined in the DRC model, involving phonological decoding or grapheme-to-phoneme conversion. During such instances, the RH may continue to contribute to visual feature processing and offer contextual assistance. However, there may be a perceptible shift in the relative contributions of lexical and sublexical processing mechanisms between the hemispheres, reflecting the dynamic nature of word recognition processes.

Several hypotheses are associated with the findings of the current study. The expertise hypothesis suggests that hemispheric functional properties arise from functional reorganization within specific regions, such as the visual word form area (VWFA), in response to perceptual learning mechanisms (Cohen and Dehaene, 2004; Dehaene et al., 2010). This hypothesis is closely linked to the concept of object familiarity, proposing that familiar objects are processed more efficiently due to extensive perceptual experience. Additionally, this hypothesis is also known as the developmental competition hypothesis (Behrmann and Plaut, 2015; Davies-Thompson et al., 2016), which posits that neural development involves competitive interactions among neurons and neural circuits engaged in object recognition. Furthermore, the expertise hypothesis is synonymous with the neuronal recycling hypothesis (Cohen and Dehaene, 2004), which suggests that brain structures originally evolved for specific functions can be repurposed, or “recycled,” for novel functions that were not part of their initial evolutionary purpose (Dehaene et al., 2010; Dehaene-Lambertz et al., 2018). These hypotheses imply the emergence of hemispheric laterality in language processing (i.e., word) and visual-perceptual processing (i.e., face) across different hemispheres. Prior studies have indicated that the LH is lateralized for language processing, as demonstrated by the dominant role of the VWFA, while the RH is lateralized for visual-perceptual processing, as indicated by the dominant role of the fusiform face area (FFA) (Canário et al., 2016; Dundas et al., 2014). These hypotheses are relevant to the observed interhemispheric familiarity effect, which occurs from the right hemisphere to the left hemisphere, as well as the intrahemispheric familiarity effect within the right hemisphere. This relevance stems from the right hemisphere’s specialization in visual-perceptual processing, which is closely associated with perceptual learning mechanisms that likely underlie the familiarity effect.

The findings of the current study have several significant implications. Firstly, it investigated interhemispheric interaction in visual word processing by employing a short SOA of 100 ms in the primed-lateralized lexical decision task. Since interhemispheric processing occurs rapidly, on a millisecond scale, the use of a brief SOA between the prime and target provides a strategic advantage in investigating the dynamic interplay between the hemispheres. Additionally, this investigation showed distinct interhemispheric interactions at an SOA of 100 ms, examining a shift from visual-perceptual processing linked to visual familiarity from the RH to the LH. This pattern of interaction highlights the dynamic and rapid nature of interhemispheric communication, occurring at millisecond intervals. Secondly, the study explored interhemispheric interaction through the lens of the familiarity effect within a repetition priming paradigm. As visual familiarity with words may be indicative of proficiency in visual word processing (Kim et al., 2022a; Kim et al., 2023b; Kuperman and Van Dyke, 2013; Mahboob, 2006), the familiarity effect serves as a valuable metric for discerning which hemisphere governs interhemispheric interactions.

While the current study yields valuable insights, there are some limitations. Firstly, the observed interhemispheric interactions may manifest more variations across narrower time intervals. Our findings suggest that the two hemispheres engage in dynamic interactions at millisecond scales, hinting at the potential for more intricate interhemispheric dynamics at even finer temporal resolutions. Broadening the spectrum of SOAs between the prime and target could examine more specific patterns of interhemispheric interaction during visual word recognition. Secondly, the duration of the prime stimulus may impact the extent of interhemispheric interactions. In the experimental task in the current study, the prime was presented for a brief duration of 50 ms. Incorporating varied prime durations within the primed-lateralized lexical decision paradigm could offer a more complicated exploration of the prime’s influence on the target. Such an approach contributes to understanding of interhemispheric interactions, examining how the activation level of one hemisphere, induced by the prime, influences subsequent processing. Thirdly, lexical decision processes may be influenced by additional cognitive factors such as attention (Kim et al., 2023a) and risk-taking propensity (Kim et al., in press). Therefore, it is crucial to explore the effects of these individual factors on parafoveal lexical decision-making to examine the interhemispheric mechanisms involved in visual word processing. Addressing these limitations in future research are expected to provide a more comprehensive exploration of interhemispheric interactions in visual word recognition.

## Conclusion

In the context of visual word processing, the current study sheds light on the intricate dynamics of interhemispheric interactions and their implications for proficiency development. By utilizing word familiarity as a guiding metric, we reveal a complicated picture wherein the RH may predominantly engage in visual-perceptual processing, while the LH may assume a greater role in sublexical/lexical processing. This hemispheric asymmetry manifests in a distinctive pattern of interhemispheric interaction, notably observed at an SOA of 100 ms. Our findings underscore the temporal intricates of these interactions, revealing the complex interplay between hemispheric specialization, familiarity, and temporal processing. Leveraging a short SOA (100 ms) in the primed-lateralized lexical decision task proves instrumental in capturing these hemispheric dynamics. In sum, our study offers valuable insights into the neural underpinnings of proficiency development in visual word recognition, highlighting the pivotal role of hemispheric interactions and the influence of word familiarity. Future investigations could delve deeper into these interactions at even finer intervals and explore the impact of varying prime durations. Such endeavors hold promise for a more comprehensive understanding of these multifaceted processes and their implications for visual word processing proficiency.

## Data Availability

The raw data supporting the conclusions of this article will be made available by the authors, without undue reservation.

## Footnotes

1 One-way analyses of variance (ANOVAs) indicated no significant differences across the four familiarity levels in terms of physical length factors [*F*(3, 296) = 2.023, *p* = 0.111 for strokes; *F*(3, 296) = 1.947, *p* = 0.122 for syllables; *F*(3, 296) = 0.202, *p* = 0.895 for morphemes; *F*(3, 296) = 0.540, *p* = 0.655 for phonemes], semantic factor [*F*(3, 296) = 0.779, *p* = 0.506 for objective meanings], and frequency factor [*F*(3, 296) = 0.408, *p* = 0.747 for log-transformed first syllable frequency].
