# Supplementary material for: Contributions of hemispheric dynamics in visual word recognition: uncovering familiarity effects through lateralized priming
Source: Front Psychol. 2024 Dec 17;15:1475475. doi: 10.3389/fpsyg.2024.1475475 (PMC11685005; doi:10.3389/fpsyg.2024.1475475)
Supplement: Supplementary file 1 [file Table_1.docx]

Appendix A

Materials

Table A in this appendix presents the Korean morphologically complex words and nonwords employed in the current study. Given that the study investigates familiarity effects using a four-level familiarity condition (F1: least familiar, F2: moderately unfamiliar, F3: moderately familiar, F4: most familiar), words are organized across four distinct columns reflecting these levels. The materials were extracted in an unbiased manner from a diverse range of categories, including newspapers (i.e., **담화는, 비상이, 우려는**), movies (i.e., **젓가락을, 형님도, 표현을**), published papers/articles (i.e., **압축은, 분포는, 원리에**), and internet blogs (i.e., **무형의, 대상에서, 지적은**). The nonwords are displayed adjacent to the final column of the word category for comparison.

| Morphologically complex words | | | | Nonwords | | | |
| --- | --- | --- | --- | --- | --- | --- | --- |
| F1 | F2 | F3 | F4 |  |  |  |  |
| 허락도 | 정책에 | 색상을 | 택시는 | 흅행에서 | 츔에 | 뮬량을 | 숩비스로 |
| 치안이 | 개인이 | 장소로도 | 국민들의 | 돋건에 | 캰과 | 쥰표가 | 늑조를 |
| 심심함을 | 자신들을 | 죽음과 | 입구에 | 츋간들이 | 댠률이 | 뱓건을 | 귿준을 |
| 도시와는 | 핑계로 | 형님도 | 최고는 | 쟙망을 | 붖해가 | 굘의 | 햡섭을 |
| 단계에서 | 희망을 | 공포를 | 핵심은 | 묠의 | 둔태로 | 컨연은 | 겯감이 |
| 선택의 | 값에 | 질서와 | 경험과 | 랸문을 | 챡인이 | 푝석에 | 굡상을 |
| 기준을 | 관련성에 | 목록만 | 꿈의 | 셜너스는 | 햑여를 | 룐일은 | 큗가락을 |
| 자본을 | 동네에서 | 모형을 | 사이의 | 톱퇴로 | 윰상과 | 읃장은 | 횸도로 |
| 시대가 | 별개의 | 외부로 | 성공을 | 즂들 | 푭스가 | 펻험에 | 슴걸음을 |
| 사전에 | 길이 | 침이나 | 여행에서 | 몁을 | 뷸정은 | 먁나는 | 냡배는 |
| 관계자는 | 결과를 | 반응은 | 영향력을 | 슘분으로 | 됻안이 | 퓹국은 | 읻형의 |
| 대중의 | 요청을 | 어휘가 | 가능성을 | 묜도는 | 먄수는 | 푠인이 | 칟심은 |
| 조직을 | 준비성이 | 인연은 | 분위기가 | 둅리에 | 챶포는 | 늉에 | 뱢미들 |
| 절차를 | 참가자는 | 재료들이 | 선배는 | 닝어와 | 먐자리를 | 겅신을 | 뇰통령이 |
| 전략을 | 현상을 | 책임인 | 지혜와 | 렫간의 | 렷력을 | 쟏정을 | 햣교를 |
| 간섭을 | 형태에 | 공감대가 | 사진을 | 렵당성을 | 묟축은 | 쳗략을 | 굔백은 |
| 전체에서 | 기름이 | 급여를 | 수준의 | 븝스는 | 픕장소를 | 뇸매의 | 댭생일이 |
| 발길이 | 땀을 | 기술에 | 자체는 | 륭청을 | 퓯도는 | 댬리를 | 뵫계로 |
| 발걸음을 | 방식으로 | 기차나 | 제품을 | 릊험과 | 햠진을 | 흗무실에 | 묩에 |
| 시도는 | 탄생일이 | 판단에 | 공기가 | 쥽습이 | 냗록만 | 귣험과 | 됵오를 |
| 빈도는 | 조례를 | 전통을 | 특색있는 | 튠란이 | 뵥려는 | 퍋포를 | 셛일이라 |
| 협력을 | 모내기와 | 단체들을 | 본인이 | 뎐계에서 | 섲공업에 | 걀통점은 | 숟에서 |
| 연설에서 | 무가 | 시기가 | 부분으로 | 쳡상을 | 믐료들이 | 랼도에 | 퍔구들을 |
| 조건에 | 준거를 | 체험과 | 비판이 | 퍚류를 | 폊위로 | 걎례를 | 뉻체에게 |
| 용어와 | 의무실에 | 힘이나 | 세상을 | 긱비를 | 갿속력은 | 뮴색을 | 낟도를 |
| 정오를 | 부스는 | 설문을 | 식탁에 | 른고는 | 켤명도 | 쳥선지를 | 캴조가 |
| 손짓으로 | 지휘부가 | 연구에서 | 차를 | 죨사로 | 옂고를 | 숃정은 | 먇언으로 |
| 사적으로 | 궤도에 | 종류의 | 내용들은 | 욛상에서 | 컥기가 | 먈청에 | 븐수로 |
| 충성심을 | 대원들이 | 개념을 | 대통령이 | 폅위기가 | 틷택의 | 헝측을 | 흊탁에 |
| 보고를 | 이득을 | 식비는 | 물건의 | 휸하철이 | 쿧비성이 | 츕문에 | 탼보에 |
| 지적은 | 휴대를 | 판매의 | 인사를 | 뇩중의 | 슏기가 | 츈박에 | 쥴를 |
| 제도를 | 제안을 | 행운을 | 일자리를 | 툳통을 | 룝인은 | 햘민들의 | 솝이나 |
| 오류를 | 저장소를 | 바닥으로 | 하나는 | 얃면으로 | 뎔닥으로 | 흘을 | 퍌림들 |
| 분류를 | 길거리에 | 기록은 | 인생의 | 갹이 | 텾이나 | 펵공을 | 뵨류의 |
| 대상에서 | 논란이 | 기운이 | 가격도 | 챳단을 | 큑가 | 슙을 | 뱐부로 |
| 유형에 | 별미로 | 수단을 | 그림들 | 몾력을 | 둠대를 | 졉상이 | 뇽련성에 |
| 설명도 | 삽시간에 | 무엇을 | 맛과 | 툽격도 | 뷴을에 | 쿕생의 | 늣락도 |
| 후자의 | 결속력을 | 봉사로 | 모습이 | 맟원들을 | 뇾유는 | 댝용을 | 퉄색있는 |
| 편법으로 | 마무리를 | 매력을 | 목적은 | 퓰록은 | 켭전에 | 켱설에서 | 줍체는 |
| 관계도 | 상관을 | 기회로 | 자리에 | 쿰전인 | 캳형에 | 큠험으로 | 뉸고를 |
| 원형의 | 소송을 | 표현을 | 검색을 | 름대가 | 춛격을 | 슐응은 | 믑혜와 |
| 모험으로 | 도박에 | 동의를 | 앞의 | 랻인으로 | 츨술에 | 죰음과 | 츙엇을 |
| 한계는 | 숙소가 | 직원들을 | 걱정은 | 킷자의 | 룹이의 | 푯적은 | 캇님도 |
| 원리에 | 필수로 | 오해가 | 결국은 | 쟘측에서 | 먑계에 | 첨기가 | 룯론에서 |
| 증인이 | 확률이 | 수고를 | 과정을 | 뵬도는 | 슉요성에 | 녕구에서 | 륩가자는 |
| 타당성을 | 횟수는 | 실험에 | 눈을 | 핒식도 | 컫거를 | 졍리에 | 툗소로도 |
| 경비를 | 단어를 | 개개인이 | 손에 | 듣이들을 | 뉼수를 | 짇행에서 | 귭계는 |
| 전역에서 | 필요성에 | 속도로 | 직장이 | 뱍력을 | 몹법으로 | 쵿품을 | 훋으로 |
| 무형의 | 분석에 | 생일이라 | 망신을 | 릅지인은 | 뭅제라고 | 켬싱함을 | 맙휘가 |
| 항목으로 | 비교를 | 공항에서 | 서론에서 | 얒형의 | 쟉차를 | 걉짓으로 | 플의를 |
| 탑을 | 사례가 | 기대감에 | 여생을 | 혇시와는 | 퍈향으로 | 냦본을 | 퓽신이 |
| 중공업에 | 서비스로 | 열정은 | 상에 | 늄과가 | 휵현을 | 뱡안이 | 귲사를 |
| 유언으로 | 양념이 | 끝에 | 행선지를 | 쟟임인 | 듭원들이 | 턈념이 | 녇례가 |
| 된서리를 | 의미를 | 껍질도 | 구조를 | 샥림자도 | 퓸태에 | 듑복이 | 븜직을 |
| 심복이 | 차량을 | 인간의 | 절감이 | 푤념을 | 륻준의 | 츶경을 | 츤시간에 |
| 재계에 | 차이점에 | 방향으로 | 탈퇴로 | 윶마가 | 뉵안을 | 븆책에 | 둡내기와 |
| 지표가 | 계기가 | 방법을 | 단위로 | 텽수를 | 겆항에서 | 횰계자는 | 햔선을 |
| 담화는 | 공통점은 | 작품을 | 보너스는 | 챧화는 | 픔인이 | 쳠길이 | 둗가에는 |
| 젓가락을 | 비용을 | 아이들을 | 안에서 | 춀무리를 | 뮨감대가 | 푣네에서 | 큽과를 |
| 차익이 | 강도에 | 환경을 | 이유는 | 큘능성을 | 갼인네만 | 쿱대를 | 킫생을 |
| 비상이 | 경호에 | 화면으로 | 정도는 | 혐름이 | 쟌대감에 | 냑호에 | 슝을 |
| 화력을 | 관측을 | 시간에 | 친구들을 | 됸단에 | 럅을 | 듖비는 | 쿋미로 |
| 행보에 | 변인으로 | 생선을 | 효과가 | 붑수에 | 득구에 | 녈회로 | 펼낌이 |
| 체제라고 | 사측에서 | 향수를 | 느낌이 | 녑용들은 | 쥼에 | 븅류를 | 둄익이 |
| 노조가 | 심신이 | 결정은 | 버스가 | 븁건의 | 셥법을 | 픅목으로 | 눕판이 |
| 시행에서 | 원장은 | 그림자도 | 노인네만 | 륨에 | 튤과의 | 륵적은 | 굗정은 |
| 광장도 | 이전인 | 계수에 | 일들 | 룍식으로 | 휻적으로 | 쵼장이 | 틉질도 |
| 압축은 | 현지인은 | 군청에 | 공간들이 | 듈개인이 | 잗가 | 팓소가 | 컂역에서 |
| 놈들한테 | 대문에 | 생가에는 | 돈으로 | 훕서리를 | 챰어를 | 횹송을 | 찯신들을 |
| 요소가 | 사건을 | 밀수를 | 공백은 | 뮥시는 | 귱득을 | 큔품을 | 힏이점에 |
| 사상과 | 사과의 | 부대를 | 지하철이 | 햗거리에 | 뮫개의 | 텹에서 | 큳도에 |
| 타격을 | 상태로 | 말을 | 내일은 | 먓간에 | 굥운을 | 븍향력을 | 퍙장도 |
| 사안이 | 수가 | 가을에 | 밥을 | 뱝운이 | 볍서와 | 졈소가 | 튭들한테 |
| 분포는 | 원인은 | 음식도 | 집에 | 츱계도 | 쥭성심을 | 슫상을 | 젼관을 |
| 우려는 | 입에서 | 소리를 | 엄마가 | 쳦차나 | 닺휘부가 | 넝체들을 | 튬형을 |

Table A. Korean morphologically complex items and nonwords used in the current study
